# Supplementary material for: Measurements of Plasma-Free Metanephrines by Immunoassay Versus Urinary Metanephrines and Catecholamines by Liquid Chromatography with Amperometric Detection for the Diagnosis of Pheochromocytoma/Paraganglioma
Source: J Clin Med. 2020 Sep 26;9(10):3108. doi: 10.3390/jcm9103108 (PMC7600173; doi:10.3390/jcm9103108)
Supplement: Supplementary file 1 [file jcm-09-03108-s001.zip › Supplementary Table and Legend to Table.docx]

| **EIA (plasma)/**  **HPLC (24h-urine)** | | **EIA (plasma)**  **sensitivity (95% CI); ULN** | | **HPLC (24h-urine)**  **sensitivity (95% CI); ULN** | | **p-value** |  |
| --- | --- | --- | --- | --- | --- | --- | --- |
| P-MN/**U-MN** | | 66.7% (53.4-77.8); 90 pg/ml | | 88.9% (77.8-94.8); 138 μg/24h | | **0.04** |  |
| P-MN*/U-MN | | 72.2% (59.1-82.4); 58 pg/ml | | 88.9% (77.8-94.8); 138 μg/24h | | 0.09 |  |
| P-MN/U-A | | 66.7% (53.4-77.8); 90 pg/ml | | 44.4% (32.0-57.6); 27 μg/24h | | 0.09 |  |
| **P-MN*/**U-A | | 72.2% (59.1-82.4); 58 pg/ml | | 44.4% (32.0-57.6); 27 μg/24h | | **0.004** |  |
| P-NMN/**U-NMN** | | 83.3% (71.3-91.0); 180 pg/ml | | 100% (93.4-100); 311 μg/24h | | **0.002** |  |
| P-NMN*/U-NMN | | 94.4% (84.9-98.5); 45-88 pg/ml | | 100% (93.4-100); 311 μg/24h | | 0.25 |  |
| **P-NMN**/U-NA | | 83.3% (71.3-91.0); 180 pg/ml | | 61.1% (47.8-73.0); 97 μg/24h | | **0.04** |  |
| **P-NMN***/U-NA | | 94.4% (84.9-98.5); 45-88 pg/ml | | 61.1% (47.8-73.0); 97 μg/24h | | **0.005** |  |
|  | |  | |  | |  |  |
| **EIA (plasma)/**  **HPLC (24h-urine)** | **EIA (plasma)**  **specificity (95% CI); ULN** | | **HPLC (24h-urine)**  **specificity (95% CI), ULN** | | **p-value** | | |
| **P-MN**/U-MN | 97.0% (95.6-97.9); 90 pg/ml | | 63.1% (59.9-66.2); 138 μg/24h | | **0.001** | | |
| **P-MN*/**U-MN | 89.4% (87.2-91.3); 58 pg/ml | | 63.1% (59.9-66.2); 138 μg/24h | | **0.001** | | |
| P-MN/**U-A** | 97.0% (95.6-97.9); 90 pg/ml | | 99.3% (98.5-99.7); 27 μg/24h | | **0.02** | | |
| P-MN*/**U-A** | 89.4% (87.2-91.3); 58 pg/ml | | 99.3% (98.5-99.7); 27 μg/24h | | **0.001** | | |
| **P-NMN**/U-NMN | 99.5% (98.8-99.8); 180 pg/ml | | 46.9% (43.6-50.2); 311 μg/24h | | **0.001** | | |
| **P-NMN***/U-NMN | 76.9% (74.1-79.6); 45-88 pg/ml | | 46.9% (43.6-50.2); 311 μg/24h | | **0.001** | | |
| **P-NMN**/U-NA | 99.5% (98.8-99.8); 180 pg/ml | | 95.2% (93.5-96.4); 97 μg/24h | | **0.001** | | |
| **P-NMN***/U-NA | 76.9% (74.1-79.6); 45-88 pg/ml | | 95.2% (93.5-96.4); 97 μg/24h | | **0.001** | | |

Table S1. Comparisons of sensitivity (above) and specificity (below) of the single plasma compared to the single 24h-urinary parameters using the ULN as summarized in Table 1. Higher sensitivities and specificities are depicted in bold. The asterisks denote P-MN and P-NMN with the corrected ULN [Weismann 2015, ref. 17].
