# Supplementary material for: Measurements of Plasma-Free Metanephrines by Immunoassay Versus Urinary Metanephrines and Catecholamines by Liquid Chromatography with Amperometric Detection for the Diagnosis of Pheochromocytoma/Paraganglioma
Source: J Clin Med. 2020 Sep 26;9(10):3108. doi: 10.3390/jcm9103108 (PMC7600173; doi:10.3390/jcm9103108)
Supplement: Supplementary file 1 [file jcm-09-03108-s001.zip › Legends to Supplementary Figures.docx]

LEGENDS TO SUPPLEMENTARY FIGURES

Supplementary Figure S1. Concentrations of biochemical parameters (in percent of the respective ULN) of patients with PGL (n=5) and PHEO (n=49). The significance of of the differences of medians were calculated by means of the Mann-Whitney U test.

Supplementary Figure S2. Post-test probability of PPGL given a positive and negative test result of studies summarized in Figure S4 on the basis of the respective pre-test probability. Please note, that 95% CI of post-TP are calculated from the numbers of the lower and the upper limits of 95% CI of sensitivity and specificity, respectively of the present study, and of [Christensen 2011, Pussard 2014, Weismann 2015]. The other six studies did not report 95% CI of sensitivity and of specificity, respectively. Thus, their reported 95% CI of post-TP is too small.

Supplementary Figure S3. ULN and diagnostic sensitivities and specificities of studies comparing plasma free metanephrines (P-MNs) by immunoassays to 24h-urinary MNs (U-MNs) and 24h-urinary catecholamines (U-CATs). One asterisk (*) denotes optimized ULN from ROC curves, two (**) the corrected ULN [Weismann 2015], and three (***) of studies that report sensitivities and specificities of single analytes only and not of the pairs.

Supplementary Figure S4. Sample size, indications for biochemical testing and criteria for exclusion of PPGL of the available literature on P-MNs by RIA and EIA. Wording of the original studies is used. ^a^ numbers sum up to the n=683 patients with ≥1 concentration above the lowest ULN of any biochemical parameter, ^b^ not further defined.
