# Supplementary figures and images for: Measurements of Plasma-Free Metanephrines by Immunoassay Versus Urinary Metanephrines and Catecholamines by Liquid Chromatography with Amperometric Detection for the Diagnosis of Pheochromocytoma/Paraganglioma
Source: J Clin Med. 2020 Sep 26;9(10):3108. doi: 10.3390/jcm9103108 (PMC7600173; doi:10.3390/jcm9103108)

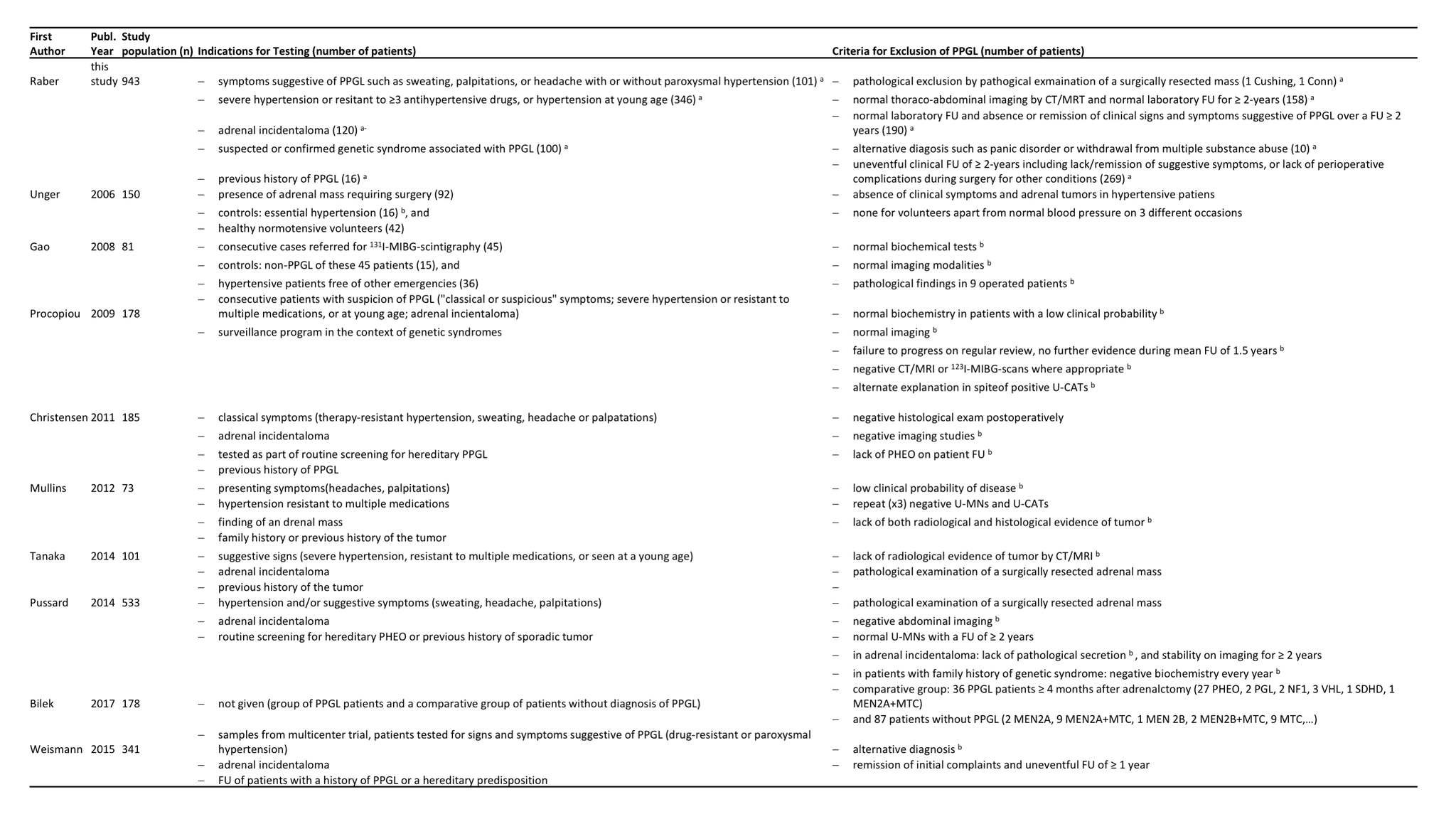

Supplement: Supplementary file 1 [file jcm-09-03108-s001.zip › Supplementary Figure S4.jpg]

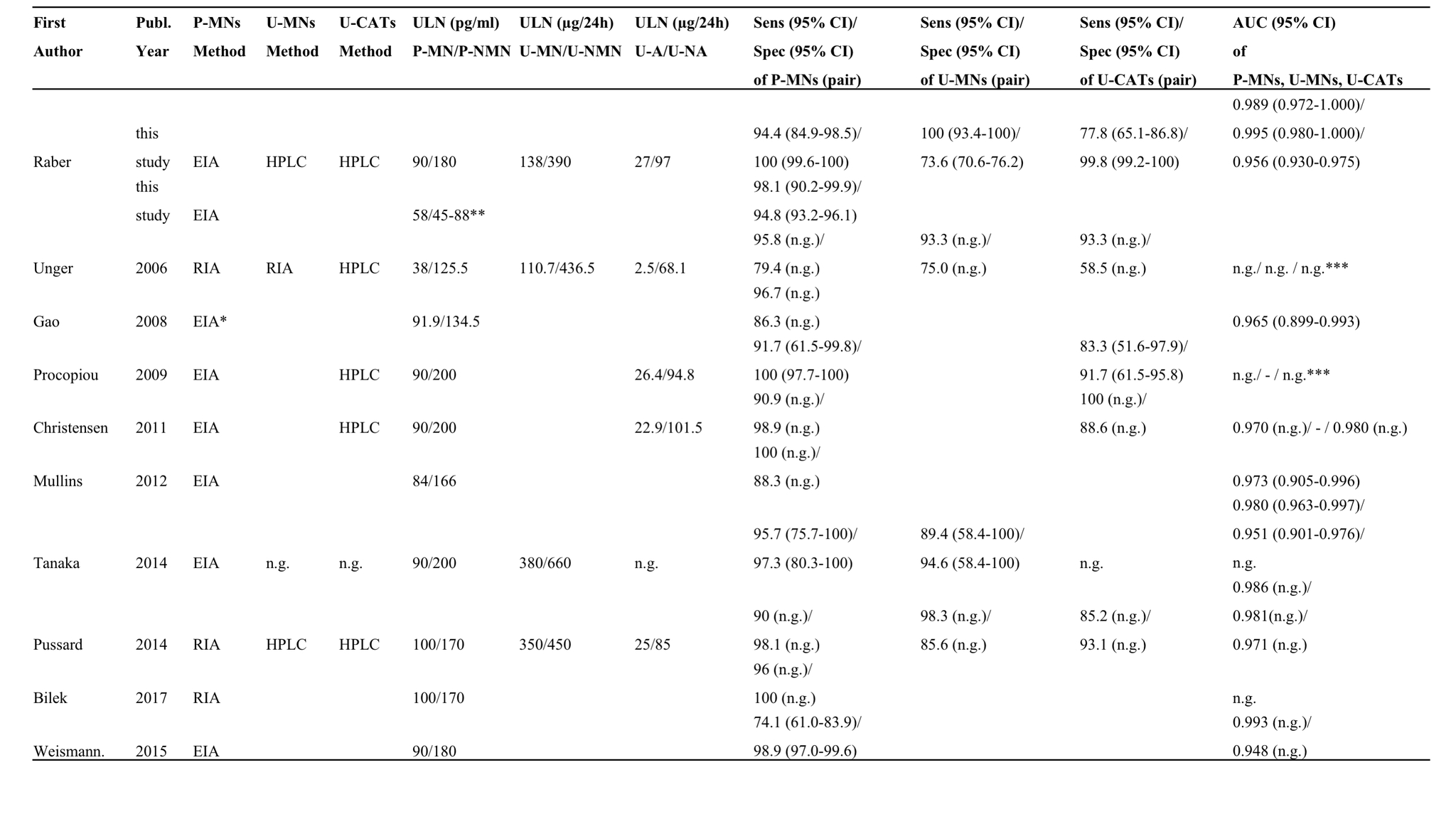

Supplement: Supplementary file 1 [file jcm-09-03108-s001.zip › Supplementary Figure S3.jpg]

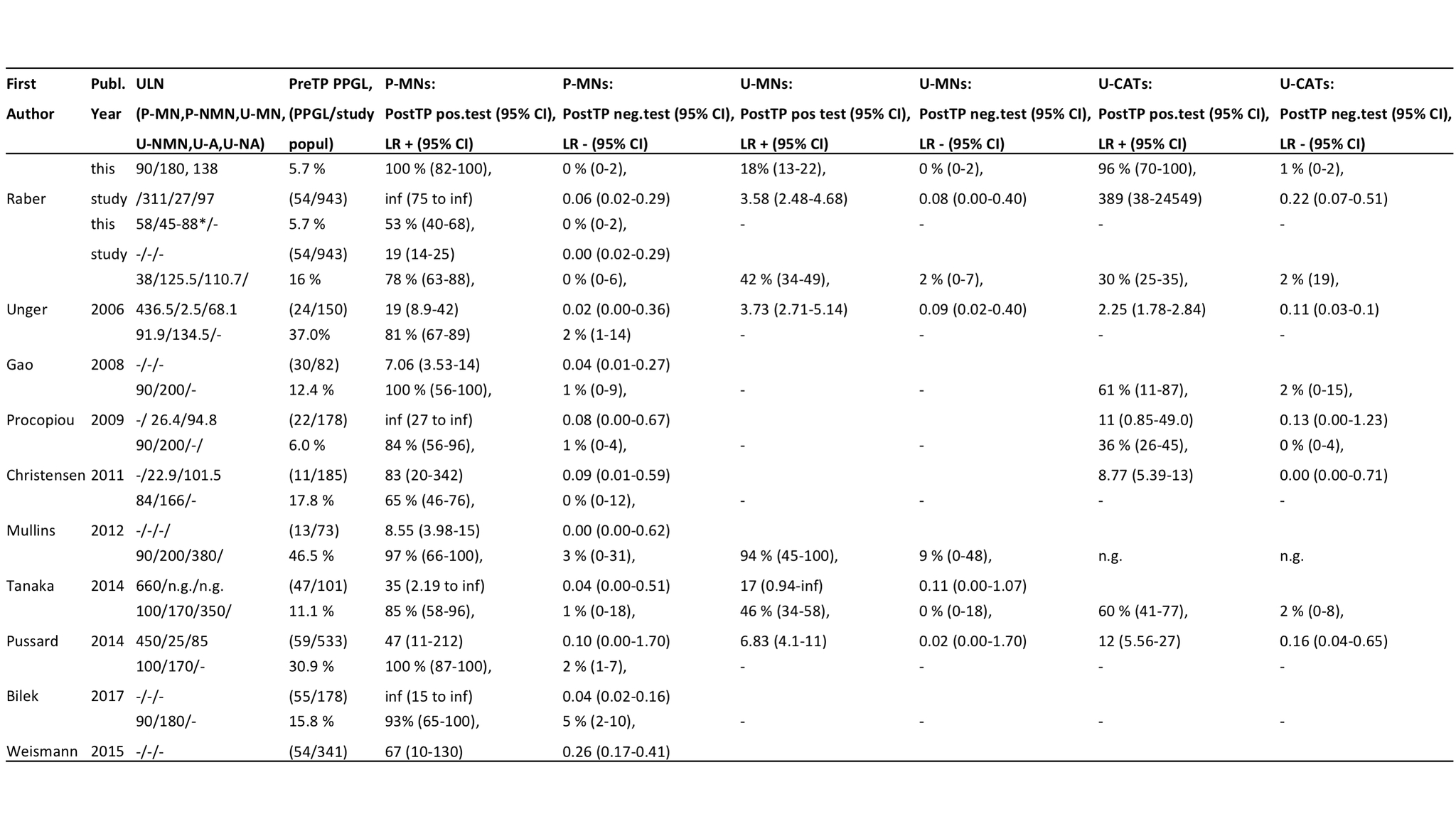

Supplement: Supplementary file 1 [file jcm-09-03108-s001.zip › Supplementary Figure S2.jpg]
